# Bilateral distal Achilles tendon sleeve avulsion: a case report

**DOI:** 10.1186/s13256-023-03892-3

**Published:** 2023-04-22

**Authors:** Illina Mohd Rothi, Mikael Åkerback, Ville Bister

**Affiliations:** 1 Department of Orthopaedics and Traumatology, Peijas Hospital, Helsinki University Hospital, Vantaa, Finland; 2 Department of Surgery, Clinicum, Faculty of Medicine, University of Helsinki, Helsinki, Finland

**Keywords:** Achilles tendon, Distal tendon rupture, Sleeve avulsion, Bilateral rupture

## Abstract

**Background:**

While Achilles tendon rupture is a common injury sustained especially in sporting events, distal Achilles tendon rupture is less common. Even rarer is a bilateral traumatic distal Achilles tendon sleeve rupture, with outcomes of such injury unknown. The following case report describes this rare injury, not reported to date elsewhere.

**Case:**

A 57-year-old Finnish man with no predisposing medical history had a traumatic bilateral distal Achilles tendon sleeve avulsion injury. Clinical and radiological evaluation confirmed the diagnosis. Treatment included suture anchors in a modified suture bridge style with customized rehabilitation protocol postoperatively. Symptoms continued to be relieved at 1 year postoperatively.

**Conclusion:**

A modified suture bridge style and meticulous rehabilitation protocol including motivated patient contributed to very satisfying results in this very rare bilateral injury.

## Background

With the increase in sports participation and the growing number of people maintaining an active lifestyle in recent times, an upward trend of tendon injuries, with Achilles tendon contributing to approximately 20% of all tendon injuries, has been observed [1–5]. The Achilles tendon is the strongest tendon in the body, with the capacity to withstand up to 12 times the individual’s body weight [6–9]. The incidence rate of Achilles tendon rupture is 7–40 per 100,000 person-years and suggested to be increasing; hence, it is the most common lower extremity tendon injury [10]. The rupture is mostly found 2–6 cm from the insertion site, otherwise known as the avascular zone [2, 6, 7, 9, 11–13]. This injury occurs most frequently in the 4th–5th decades, being twice as common in males, following a sporting activity [6, 8–10]. Less commonly, the tendon can be ruptured distally at its calcaneal insertion, either as an avulsion fracture with a bony piece from the calcaneus or as a continuous sleeve avulsion rupture without any bony fragment [1, 2, 6, 14]. This complex injury poses a tricky clinical management issue since there is minimal tissue to assist in direct repair of the tendon onto the insertion site [6]. Generally, patients who present with this injury have specific risk factors, for example, insertional Achilles tendinopathy, tendon degeneration, calcific spur formation, steroid therapy, or small transverse microtears, which over time may progress to complete tears [2–7, 13–15].

Even less common is the occurrence of simultaneous bilateral ruptures, with a reported incidence of < 1% overall [8, 9]. These are usually mid-tendon ruptures with predisposing risk factors. A few case reports have been published on traumatic bilateral Achilles tendon ruptures without risk factors [8, 10]. A case report in 2016 described such an incident and demonstrated the use of ultrasonography in diagnosis [8]. However, bilateral sleeve avulsion of Achilles tendon is exceptionally rare, and to our knowledge, this is the first reported case of simultaneous bilateral sleeve avulsion and its treatment, follow-up, and recovery.

## Case details

**Fig. 1:**
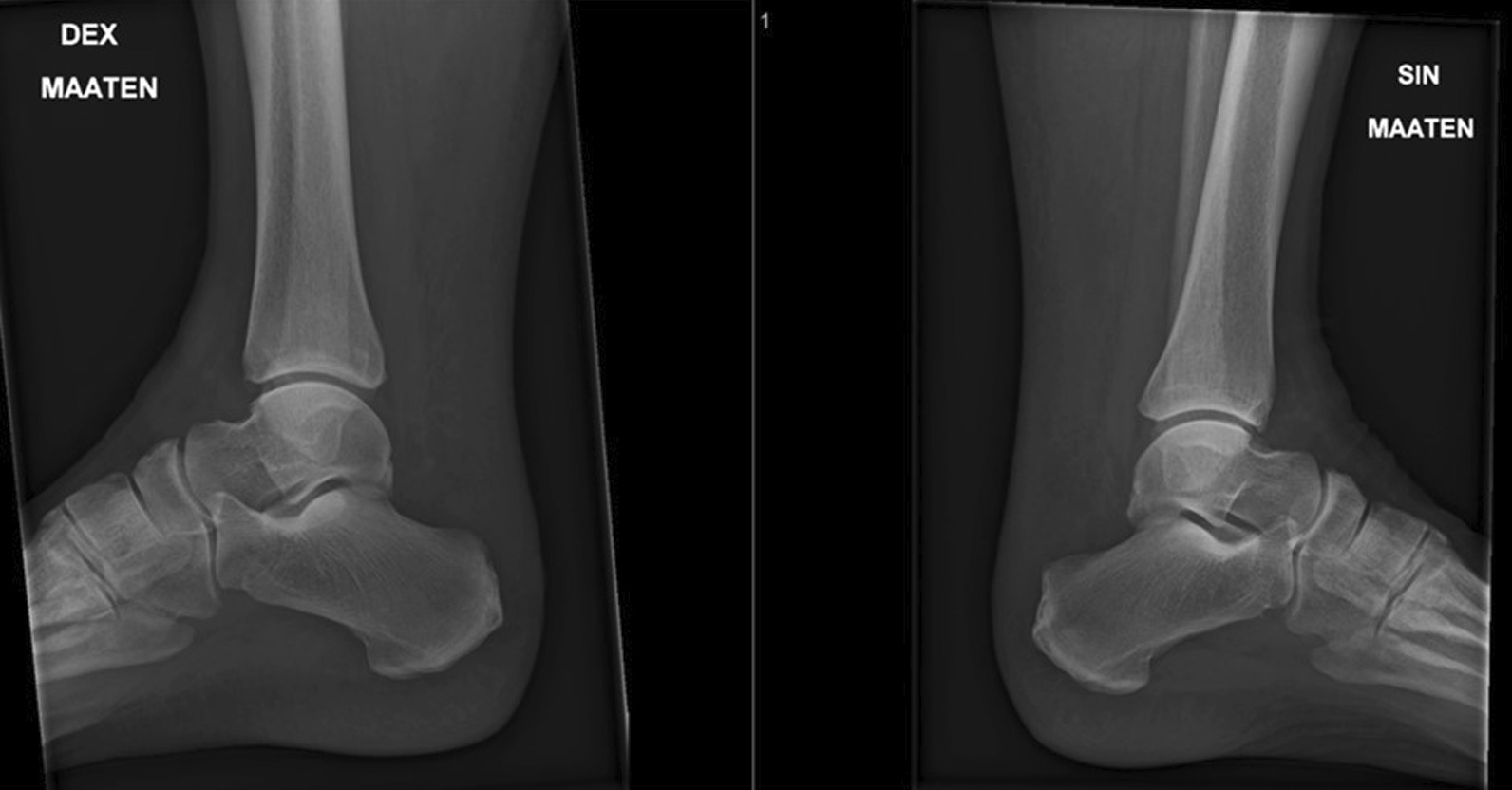
X-rays showing no bony traumas on either side

**Fig. 2:**
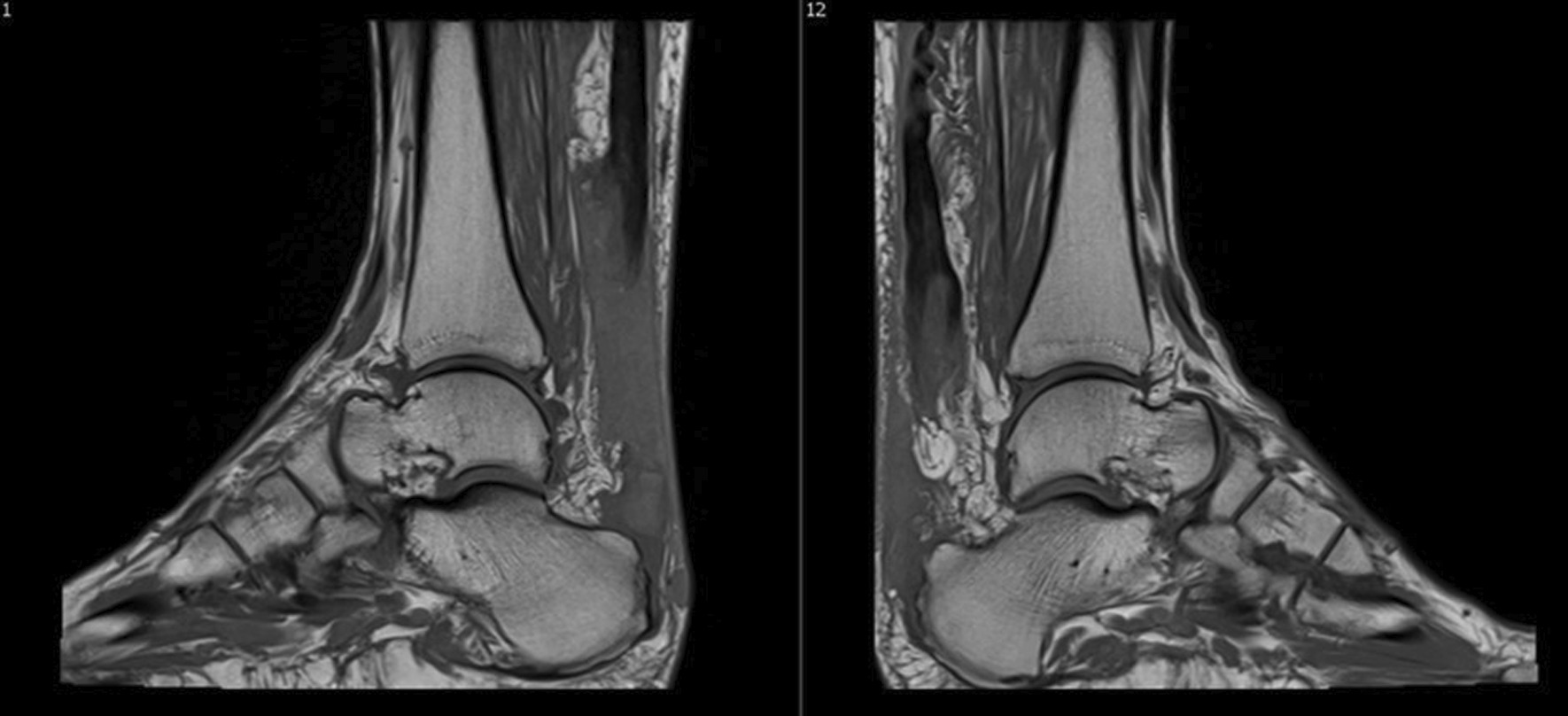
Magnetic resonance imaging showing complete avulsion of the Achilles tendons from calcaneus insertions

A 57-year-old Finnish male patient presented with a history of high blood pressure and obesity (116 kg, BMI 43) and two gastrointestinal surgeries (no malignancies); otherwise, no chronic illnesses. He had no previous injections of cortisone or administration of fluoroquinolone-type medication, but symptoms were present in the Achilles tendon area bilaterally, which were treated in a conservative manner. No previous imaging [magnetic resonance imaging (MRI) and so on]. The ankles were fit to perform sportive activities. The patient was playing badminton and charging the net when he suddenly heard and felt two consecutive snaps from the ankles. He was transferred to the Helsinki University Hospital Emergency Department, and after clinical and radiological evaluations (Fig. 1), both ankles were further examined with magnetic resonance imaging (MRI). The diagnosis was bilateral sleeve avulsion of the Achilles tendon (Fig. 2).

**Fig. 3:**
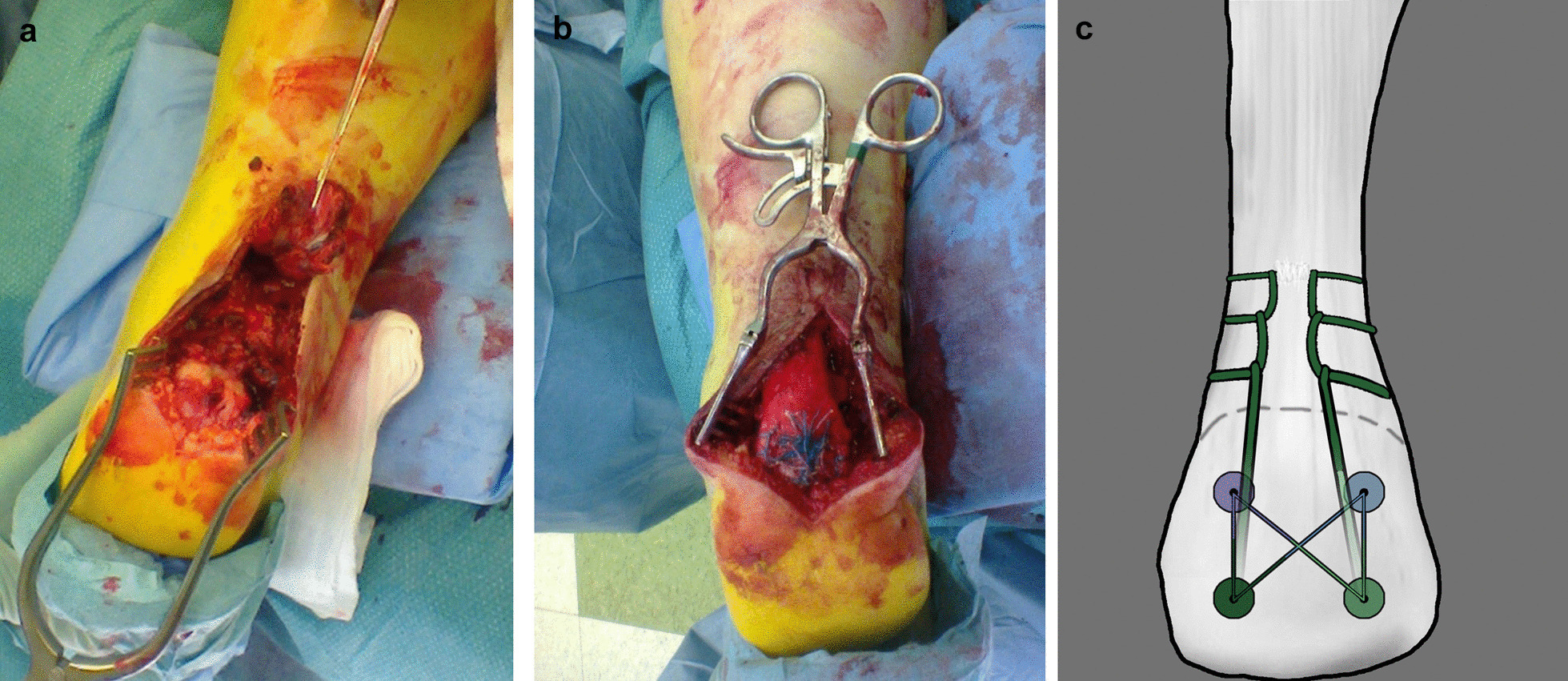
**a** Left ankle after exposure. **b** Left ankle after fixation

Both Achilles tendons were operated on in the same session 3 days after the injury using suture anchors [JuggerKnot (left ankle) and FASTak (right ankle)] with a modified suture bridge style. Four anchors were placed in the figure of a square, but the lower anchors used Krackow sutures to get larger purchase from the tendon. The proximal anchor sutures were pulled through the tendon and used as a more classical style of suture bridge with the tails of lower suture anchors (Fig. 3a–c). The postoperative rehabilitation protocol is presented in Table 1.

**Table 1:** Postoperative rehabilitation protocol

Time	Weight bearing	Support	Range of motion and strength training
0–3 weeks	non-weight bearing	walker boot + wheelchair	up to pain limit
3–6 weeks	full weight bearing with heel lifts	walker boot	
6 weeks	toe raising with half weight, one-leg training was encouraged when the patient felt he could tolerate it	normal shoe with heel lifts (1–5 cm)	
8 weeks			swimming and bicycling with heel lifts
16 weeks		normal shoes without heel lifts	
9 months			full power including jumping

**Fig. 4:**
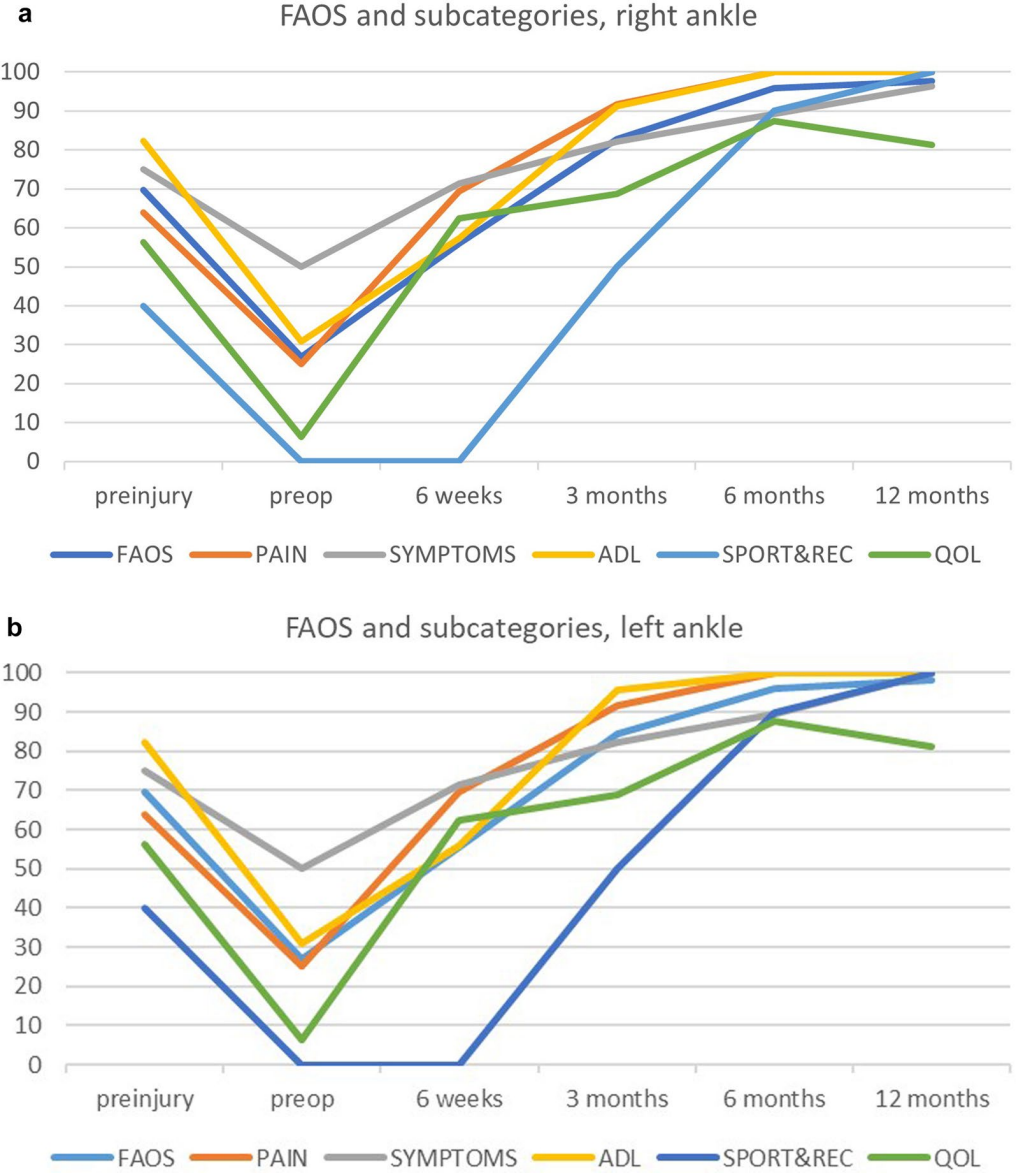
**a** Foot and Ankle Outcome Survey results for right ankle. **b** Foot and Ankle Outcome Survey results for left ankle

**Fig. 5:**
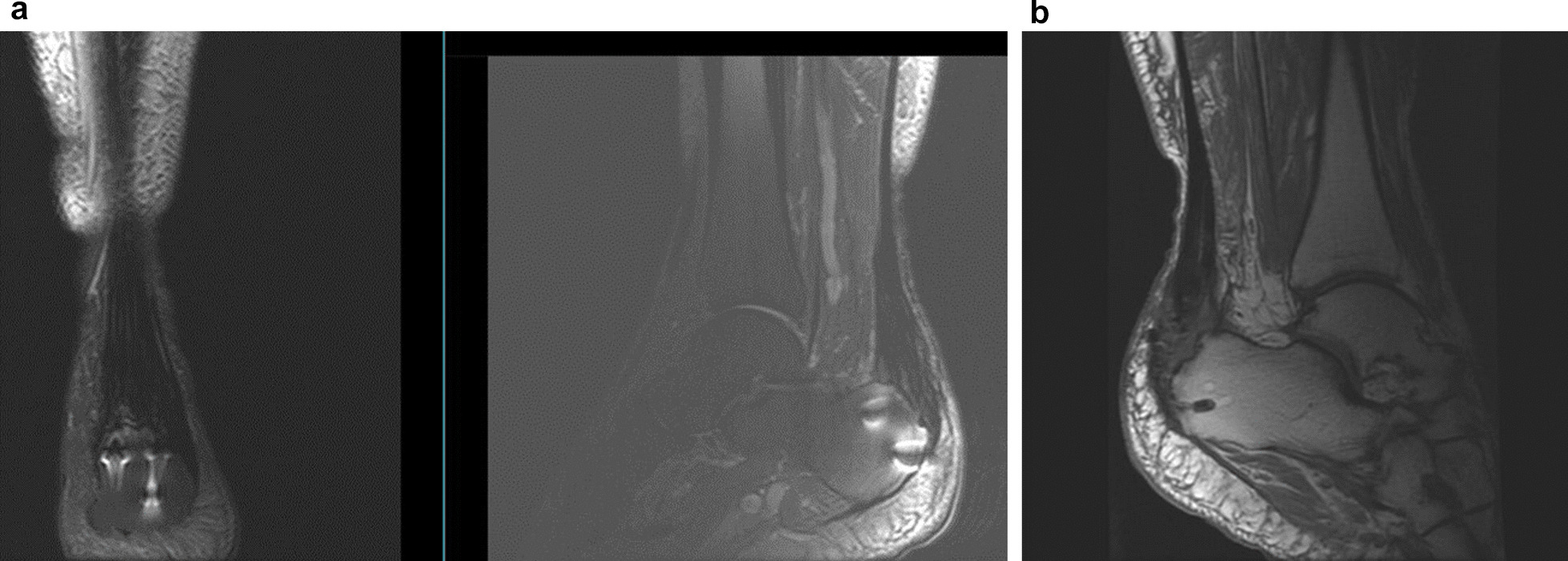
**a** Magnetic resonance imaging from the right ankle 12 months postoperatively. **b** Magnetic resonance imaging from the left ankle 12 months postoperatively

The patient filled in Foot and Ankle Outcome Survey (FAOS) questionnaires evaluating preinjury levels (naturally posthumously relying on his memory 2 days after the injury), preoperatively (2 days after the injury/1 day before the operation), and then during every follow-up visit at 6 weeks, 3 months, 6 months, and 12 months, evaluating both ankles separately (Fig. 4a and b). The right side had problems with wound healing and wound revision was performed bedside 3 weeks postoperatively by administering negative pressure wound therapy (NPWT). Both wounds were healed at 12 weeks postoperatively. At the 6-month follow-up, the patient was quite satisfied and pain free. At the 1 year follow-up—the final control visit—the patient felt satisfied with the outcome and was symptom free, but was unable to return to running and jumping activities due to fear of re-injury. Otherwise, the rehabilitation was a success and formatted to everyday routine. At this follow-up, our skilled physical therapist/research assistant performed American Orthopedic Foot and Ankle Society (AOFAS) measurement and additional testing. Both ankles were also evaluated with MRIs (Fig. 5a and b).

AOFAS score was 100 on the right side and 97 on the left side (hindfoot motion slightly decreased). QoL (quality of life) was limited to 81 because of the changes that the patient chose to make to avoid possible re-injury. FAOS score was 98 in both ankles, and improvements were noted in every subcategory; all of them were better than before the injury (Fig. 4a and b).

The patient could do repetitive toe raises up to 38 repetitions on the right and 44 repetitions on the left side. The circumferences of the calves were 45/45 cm, respectively, and 5 cm proximally from the ankle 23/24 cm, respectively. There was no need to change shoes or occupation because of the injury.

After the follow-up visit, the patient was encouraged to increase the workload to get back to the activities that were important for QoL preceding the injury.

The patient was contacted 5 years after the injury. He remained very satisfied with the result and reported no pain or symptoms. He has continued rehabilitation training and perceives his ankles to be in better shape than before the injury. He also lost 8 kg during the rehabilitation period. He is able to play golf and run, but, unfortunately, he has given up badminton.

## Discussion and conclusions

A literature review yielded no similar cases. Articles that have been written on this topic concern unilateral incidents. Previously, the decision on how to manage this injury was divided into nonoperative versus operative cases, especially between the general population and athletes given their different demands, with no general consensus of a “standard” treatment technique [1, 5, 13]. Later on, the results have shown that both groups benefit from operative treatment, with the advantages far outweighing the risks [15]. Abundant research has been conducted over the years to optimize the repair of the distal Achilles tendon surgically, using titanium anchors, transosseus drilling, whip stitch technique, or V–Y plasty if necessary [4, 6, 7, 11, 13, 16]. Within the last 3 years, however, there has been a move toward less invasive techniques to overcome some of the risks of surgery, especially wound breakdown or infection [2, 3, 12, 17]. These techniques involve small incisions over the site of surgery averaging 3–5 cm longitudinally, with one study describing use of ipsilateral semitendinosus autografting to restore continuity of the tendon [3]. When combined with ultrasonography guidance, improved visualization is achieved with minimal wound length [2].

Most earlier studies have had a follow-up period of 2–5 years, with the general consensus showing improvement to preoperative scores [2, 4, 7, 12, 13, 15, 18]. The AOFAS score was most commonly used, with all studies showing significant improvement postoperatively, except when linked to patients with higher BMI (body mass index); in that case, the scores were actually worse [4]. The functionality outcomes of the suture anchor technique focused on postoperative tendon elongation and end-range flexion weakness, with no visually altered gait found in participants [1]. Patients were generally able to return to full sporting activity by 9 months postoperatively with this minimally invasive technique; that is, they had a shorter and earlier rehabilitation period and improved ankle function recovery with a full return to sports [18]. Heel pain also seems to continue to decrease over time to complete resolution [14].

Our patient had a marked improvement in FAOS score, attaining a level that was even better than before the injury. The patient had a history of Achilles tendon symptoms, and thus, the postoperative comprehensive and meticulous rehabilitation may have had a huge impact on symptoms and performance. The major improvement, with clinical significance, occurred during the first 3 months (ADL and pain) [19], but because of residual symptoms sportive activities as well as quality of life suffered, which nevertheless continued improving at least up to 6–12 months postoperatively. It might be that the injury had an impact on how the patient handles difficult situations, and he learned to cope with them better, leading to evaluating possible residual symptoms as less uncomfortable.

The patient and his surgeons are very satisfied with the result of treating this rare injury pattern with modified suture bridge style anchoring and meticulous rehabilitation.

## Data Availability

The datasets used during the study are available from the corresponding author on reasonable request. All data generated and analyzed during this study are included in this article.

## Abbreviations

ADL: Activities of daily living
AOFAS: American Orthopedic Foot and Ankle Society
BMI: Body mass index
FAOS: Foot and Ankle Outcome Survey
MIC: Minimal improvement change
MRI: Magnetic resonance imaging
NPWT: Negative wound pressure therapy
QoL: Quality of life
